# Somatisation: illness perspectives of asylum seeker and refugee patients from the former country of Yugoslavia

**DOI:** 10.1186/1471-2296-7-10

**Published:** 2006-02-15

**Authors:** Noelle Junod Perron, Patricia Hudelson

**Affiliations:** 1 Medical Outpatient Clinic, Department of Community Medicine, Geneva University, Hospitals, Switzerland

## Abstract

**Background:**

Somatisation is particularly challenging in multicultural contexts where patients and physicians often differ in terms of their illness-related beliefs and practices and health care expectations. This paper reports on a exploratory study aimed at better understanding how asylum seeker and refugee patients from the former country of Yugoslavia who were identified by their physicians as somatising make sense of their suffering.

**Methods:**

We conducted semi-structured interviews with 26 asylum seeker and refugee patients from the former country of Yugoslavia who attended the general medicine outpatient clinic of a Swiss teaching Hospital and were identified as presenting with somatisation. Interviews explored patients' illness perspectives and health care expectations. Interviews were audio taped, transcribed verbatim and analyzed to identify key themes in patients' narratives.

**Results:**

Patients attributed the onset of symptoms to past traumatic experiences and tended to attribute their persistence to current living conditions and uncertain legal status. Patients formulated their suffering in both medical and social/legal terms, and sought help from physicians for both types of problems.

**Conclusion:**

Awareness of how asylum seeker and refugee patients make sense of their suffering can help physicians to better understand patients' expectations of the clinical encounter, and the particular nature and constraints of the patient-provider relationship in the context of asylum.

## Background

In primary care, 10–35% of patients present physical symptoms that appear to be unrelated to organic pathology [1-3]. These may be variously referred to as "medically unexplained symptoms", functional somatic symptoms or somatisation, reflecting the degree to which such symptoms are understood as an abnormality in the physical functioning of the body that can not be detected in terms of observable structural changes or as problem of psychosocial etiology [4]. Regardless of the term used, however, most researchers and clinicians consider such symptoms to be a reflection of psychological and emotional problems [5,6].

Somatisation is challenging because physicians and patients often disagree about the causes, meaning, severity and treatment of such symptoms[7,8]. Somatisation can be particularly challenging in multicultural contexts where social, cultural and linguistic differences between patients and physicians can create additional barriers to communication and to development of a therapeutic alliance with the patient [9,10].

In Geneva, Switzerland, nearly 40% of the population is foreign born, and physicians frequently encounter difficulties caring for patients from diverse social and cultural background. Two studies conducted at the general medicine outpatient clinic at the Geneva University found that physicians felt particularly challenged by somatising immigrant patients [11,12]. They described them as unable to verbally express their emotions or to make the link between psycho-social events and physical symptoms, and wondered whether patients' cultural beliefs prevented them from accepting the psychological basis of their symptoms. The physicians were also frustrated by what they felt were patients' inappropriate and unrealistic expectations of physicians and of the health care system. These physicians felt particularly unable to bridge the cultural and communication divide they experienced with asylum and refugee patients from the former country of Yugoslavia who presented with somatisation.

The purpose of the current study was two-fold: to gain insight into the illness related beliefs and health care expectations of asylum and refugee patients from former Yugoslavia who had been identified by their physicians as presenting with somatisation; and to provide information that could be incorporated into junior doctors' training activities aimed at strengthening their ability to care for socially and culturally diverse patients.

## Methods

### Setting

In Switzerland, asylum seekers and refugees represent 1.3% of the total population living in the country. Most come from Africa, the Middle East and the Balkans. On average, 22 000 people have applied every year between 2000 and 2002. The asylum process can last up to several years after which only 6.4% of asylum applications are accepted. While awaiting a decision, asylum seekers receive welfare benefits (considered to be relatively high compare to other European countries) and basic medical insurance [13]. They are housed initially in shared rooms or dormitories and then generally move to small, independent apartments. Children attend school and adults may seek employment after 3 months.

The study was conducted at the outpatient clinic of the Community Medicine Department, which is part of the Geneva University Hospitals, Switzerland. The clinic (referred to locally as the Polimed) conducts approximately 12 000 scheduled and 12 000 unscheduled consultations per year with patients over the age of 16 years. In 2002, refugees and asylum seekers made up approximately 30% of scheduled consultations. A pool of trained interpreters is made available free-of-charge to patients. Care is provided by 15–18 junior doctors who spend one year working at the Polimed as part of their training in general or internal medicine.

### Study design and data collection

The interviews began with an open-ended question aimed at eliciting the patients' illness narrative ("Please tell me about your illness"). This was then followed by a series of probes aimed at exploring the patients' perceptions of the cause, severity and expected evolution of the illness, the impact of the illness on the patient's life and any illness-related worries, the patients' coping strategies, and the patient's health care expectations. All interviews were conducted by the same person (NJP) in French, with the assistance of professional medical interpreters. Interpreters were instructed to translate word-for-word when possible, and to indicate when they encountered difficulties or confusion. Interviews generally lasted 90–120 minutes. The study was approved by the Geneva University Hospitals Research Ethics Committee.

### Study population

Fourteen junior doctors working at the Polimed in 2002 were presented with a list of all patients they had seen by appointment during the previous 6 months and were asked to identify which patients they considered to be somatisers. We excluded two other doctors who started to work only a few months before the study started because we felt that their experience with patients was too short. Although we did not provide the doctors with a definition of somatisation, a previous study at the Polimed found that junior doctors generally defined somatisation as the presence of vague, repetitive symptoms in the absence of evidence of objective physiological or biological functioning [11]. They also all considered somatisation to be the physical expression of psychological or social distress or as a sign of underlying psychiatric morbidity [11]. In this study, we were interested in exploring the illness perspectives of patients who were identified by their doctors as somatisers and therefore difficult to care for. Ten percent of their patients were identified as somatisers, with the largest proportion coming from the former country of Yugoslavia (41%, n = 72) and having a refugee or asylum seeker status. Since junior physicians considered these patients to be particularly challenging, we decided to focus our study on this population. A review of the medical files revealed that most of the patients from former Yugoslavia described as somatisers had also diagnoses of depression, anxiety and/or post-traumatic stress disorders. Only a small minority were exclusively diagnosed with somatoform disorders.

From the total list of 72 patients, we interviewed a convenience sample of 26 patients. Patients considered by their physicians to be too fragile to undergo interviews where past trauma might be evoked were excluded (10), as were patients with invalid addresses and telephone numbers (7). We invited a total of 31 patients to participate in the study; 5 patients declined because they associated our interviews with asylum hearings. Written, informed consent was obtained through professional interpreters in patients' native language.

We compared sociodemographic characteristics of participants and non-participants and found that while participants were slightly younger (mean 36 vs 41 years) and more came from Kosovo (54% vs 28%), there were no significant differences between participants and non-participants in terms of sex, religion, place of residence, legal status, length of time in Switzerland, length of follow-up at the outpatient clinic and psychiatric co-morbidity.

### Qualitative analysis

Interviews were tape-recorded, and translators' utterances were transcribed verbatim. No back translation was conducted.

Analysis of transcripts followed an "editing style" approach [14], and focused primarily on identifying patients' perceptions of the cause, severity and expected evolution of the illness, the impact of the illness on the patient's life and any illness-related worries, the patients' coping strategies, and the patient's health care expectations. Both authors read each transcript, identifying key passages which corresponded to the topics of interest. The authors then discussed each transcript, comparing and contrasting their observations and developing codes. Once consensus was reached on an overall coding scheme, NJP coded all interviews using Winmax software for qualitative analysis [15]. Coded segments were then reviewed by PH, and any differences in opinion about coding were then discussed and resolved. Data display tables were then created to facilitate the examination of similarities and differences across respondents to identify key themes.

## Results

Twenty six patients participated in the study. Most of them were female asylum seekers from Bosnia and Kosovo, living with family members in rented flats (See Table 1 for patient characteristics). The main complaints were headaches (20/26), fatigue (12/26), bone and joint pains 17/26), nervousness (18/26), sleep disturbances (7/26) and anxiety (12/26).

**Table 1:** Socio-demographic characteristics of study participants

Mean age in years (SD)	36 (+/- 10)
Female (N)	20
Male (N)	6

Kosovo (N)	14
Bosnia-Herzegovina (N)	11
South Serbia (N)	1

Muslim (N)	26

Asylum seekers (N)	23
Refugees (N)	3

No professional activity (N)	25
Professional activity (N)	1

Lives in independent apartment (N)	19
Lives in shared rooms/dormitory (N)	7

Lives with family members (N)	24
Other (N)	2

Mean number of years in Switzerland (SD)	3.2 (+/- 2.3)

Mean number of years attending the outpatient clinic (SD)	2.31 (+/- 1.5)



### The role of pre-migration experiences in explaining illness

Most patients (19/26) made a link between present symptoms and past traumatic experiences. Traumatic events such as war, flight and loss of loved ones were often mentioned to explain how and why their symptoms started.

≪ It was the war, when we were fleeing...we suffered a lot. Because I didn't have pain like this before...And then there were members of my family that were killed as well. There's an underlying nervousness that....yes, I have stomach pains and my body trembles and I'm exhausted..." (P9)

≪ It's the war. We are no longer in good health and in the head we're no longer normal. This is all since the war. ≫ (P13)

However, even patients who did not attribute their health problems traumatic events reported that their symptoms worsened when they thought about the past.

"But the epilepsy comes more often when I think..[When you think, what kind of thoughts do you have?] ...about the past..."(P2)

"When I first arrived, I had headaches all the time. Now I have them less often. [When do you get them?] It hurts the worst when I think about something bad, when I think about the past...I tell myself all sorts of things in my head and then I start to hurt." (P7)

While most patients believed that past traumatic experiences were the cause of their present symptoms, they did not feel that focusing on those experiences would alleviate their suffering. They tended to believe that "what's done is done" and that one had to learn to live with it.

≪ You don't suffer like that for no reason. I'm disturbed by what happened. I'm disturbed because I think about my husband. He was the man of my life. Life goes on, one has to live, but even so the memories come back...I can't do anything...what happened has happened ≫. (P6)

Even though patients made an association between past traumatic experiences and current symptoms, this did not preclude belief in a physical cause of their suffering as well. Many patients were convinced that their pre-migration experiences had caused some sort of physical damage, and that this physical damage was the true underlying cause of their symptoms.

"I was destroyed as a result of the past, and then the illness made me worse". (P1)

Patients sought to identify and treat the underlying physical cause of their symptoms, and for five informants the absence of organic findings fueled their fears about serious illness, functional disability and even death.

≪ If I didn't have something, I wouldn't hurt. But they tell me "you're fine." I don't know what to say ... ≫ (P9)

≪ I don't know what I have. They did tests, they don't know what I have...What worries me the most is that I'm young, and I'm afraid, and I'm always thinking that I'm going to die..." (P2)

### The role of post-migration experiences in explaining persistence of symptoms

While patients talked about pre-migration experiences as the underlying cause of their illness, they talked about current life conditions as perpetuating their symptoms and posing barriers to improvement. They felt that financial worries (4/26), worries about their children (7/26), uncertainty about the future (14/26), fear of expulsion (14/26), and lack of social support (7/26) made them vulnerable to chronic and worsening physical and psychological symptoms.

"When I start to think again, I think about all the problems around me. My past, my children, what happened to me, what could happen to me...What's going to happen with my life, the life of my children, what they're going to do in life...And then it starts again, I feel bad, I have pain. ≫ (P17)

≪ Fear is always present. Especially when you're at home, when you don't have any work, when you have lots of worries...such as how to feed and school the children. It's so many things...and it's clear that then you hurt all over" (P23)

### Medical care as an answer to both physical and social problems

Patients believed their symptoms were caused by "real" or organic problems, even if not yet identified, and that post-migration social conditions contributed to their chronicity. As a result, patients sought both medical and social solutions to their suffering. Patients put much emphasis on being examined, doing tests (14/26) and receiving medical treatment (23/26) but also felt that their well-being depended on resolving their practical social problems.

≪ What should be done to improve your health?" "First, get legal status here. Or at least receive a clear response: 'You can stay' or 'You cannot stay'... at least that way I can know." (P24)

≪ How did you feel when you got your B permit (legal status)? " "I felt relieved...which is totally normal...I told myself I'm going to stay here for awhile because my children are going to finish school here, and they won't be pulled apart in terms of schooling...that made me feel a lot better" (P17)

Interestingly, some patients looked to physicians to help them resolve their social problems (6/26). While they did not consider this to be a typical or normal part of a physician's role, they recognized that physicians could potentially provide medical certificates that could help in their request for asylum.

"I don't want to be expelled, I don't want to suffer anymore...I have two friends, they got the B permit (legal status) and say that they got it through their physician." (P9)

Many patients described their isolation and lack of social networks (14/26), and appreciated the openness, respect and understanding shown to them by physicians. While social talk, counseling and expression of emotions was not generally viewed or valued as medical treatment, it was positively experienced as legitimizing their suffering.

"They were interested in me and really took care of me. They told me "whatever you need, we can help you, we can help you...whatever documents you need, we can help you because we know that your life is difficult." (P1)

"She [the doctor] gives me the strength to go on. She tells me "you're going to get better, your wishes will be fulfilled little by little". She says "Little by little you will get better. You need to take classes to learn French. In that way you won't spend all your time thinking about what's happening at home. Sometimes it helps to go out, breathe fresh air..." (P26)

≪ I hoped to be listened to, and really that's what I found because I talk about my worries and [the doctor] listens to me and gives me advice. Basically, he's the only person I can confide in and talk to." (P21)

Only a few patients (5/26) did not enjoy talking, especially when discussions focused on the past.

"I don't like the appointments (with the doctor). I have to describe everything that happened, and how it happened. Afterwards, I feel sick for two weeks" (P3)

In contrast with their experiences at the outpatient clinic, patients' pre-migration health care experiences were characterized by high cost, brief consultations focused on blood tests and medications, poor patient/provider communication and a fear of being labeled as mentally ill.

"I feel good here, because they listen to me, and I can tell everything I have to tell. Back home when we went to the doctor, the doctor didn't even look at you – he just wrote a prescription and that was it. People didn't even greet each other the way they do here when you enter the doctor's office. They didn't even say good morning or good evening or goodbye...the doctor would just ask where you hurt, he would write a prescription and that's it. You didn't talk...whereas here you always have an hour to talk. And back home you always had to bring some sort of bribe for the doctor..." (P10)

≪ What did you do to try to get better (back in Kosovo)?" "I didn't do anything because if I had gone to what we call a neuropsychiatrist I would have been immediately labeled as crazy. Here, it's good, there's a totally different approach." (P5)

Only a minority of patients (3/26) felt that because of their legal status, they did not have the same access to care and did not deserve the same attention as other people.

"When I come to the hospital, I want to have more tests done or at least be hospitalized to do everything, to find out what I have. I asked my doctor. But maybe because I'm a refugee they don't do that". (P2)

## Discussion

Our results indicate that study participants perceived a causal link between their present illness and their pre-migration traumatic experiences. They attributed persistence of their symptoms in part to the difficulties of their current living conditions and their uncertain legal status. However, the fact that patients established a link between their psychological and physical suffering did not preclude their desire to seek medical diagnosis and treatment for their symptoms. Patients believed that their migration-related experiences could cause physical disease or dysfunction, requiring timely investigation and appropriate treatment. Nonetheless they appreciated that physicians helped them with both physical and social/legal problems.

Our finding that somatising patients tended to attribute their suffering to both physical and social causes and to perceive answers and healing strategies outside themselves are consistent with previous studies [16-18]. Other studies have also found that such patterns of response may be reinforced in an immigration context [19-21]. Several studies in different countries show that some immigrants are more prone to develop somatisation than other groups of people and seek more help from primary care physicians than from mental health professionals [22,23].

The importance of post-migration stressors is supported by studies conducted among refugees communities that demonstrated that distress among refugees persists because of psychosocial maladjustment, lack of social support and acculturation difficulties [24-26]. Separation from family, loneliness, fears of being sent home, poverty and discrimination are considered to negatively influence the mental health of asylum seekers [27,28].

Our findings suggest also that both previous health care experiences in their home countries and present health care conditions encourage patients to focus on physical symptoms and expect medical and technical solutions to their problems. On the one hand, patients in our study reported that health services in their home country did not generally address psychological distress and that they feared being labelled as mentally ill. Anthropological studies suggest that such situations may incite patients to emphasize physical symptoms of distress [10,18]. On the other hand, immigrants' free and easy access to high-tech care and a traditionally biomedical approach to health problems in Switzerland may also create high expectations towards medical care and further encourage the physical expression of suffering. This is supported by previous research which shows that the expression of distress through either somatic or psychological symptoms is shaped by the availability, accessibility and structure of the primary care system [18,29].

At the same time, most patients in our study complained of social isolation and appreciated the psychological and social support they received from their physicians. The fact that they experienced such support almost exclusively in the medical setting may further contribute to the medicalisation of their suffering and encourage them to consider illness as a means to "articulate their social suffering, express their unsatisfactory integration and legitimate their demands for improved living conditions in the host country" [30]. In our country as in many other countries of Europe, only a limited number of asylum seekers are admitted as refugees after many years of procedures. Swiss asylum authorities sometimes rely on medical certificates to make decisions about asylum applications. Because asylum seekers whose medical conditions cannot be adequately treated in their home country can obtain a prolongation of their stay in Switzerland, it may be that the asylum process incites people to frame distress as a medical condition [31]. The asylum context shares many similarities with other contexts of care where social, political and medical issues are closely interlinked (use of post-traumatic stress disorder diagnosis for US veterans and compensation system [31,32]; low back pain or somatoform disorder and claims for invalidity pensions [33]. In such contexts, the doctor is generally seen not only as a source of health care but also as a gatekeeper or facilitator to social acceptance, financial or legal benefits.

The medical setting and physician status of the interviewer may have influenced the content and emphases of patients' narratives as well [34,35]. If participants in our study perceived the study interview as another means to legitimizing their suffering and asylum request, their illness narratives may have emphasized what they believe to be of most interest or importance to asylum authorities.

Our study has several weaknesses. We interviewed a convenience sample of patients who may not be representative of all Kosovar and Bosnian patients considered to be somatisers, and the small sample size did not allow us to explore potential differences between asylum seekers and refugees. We also did not check the accuracy of interpreters' translations, although we did use professionally trained medical interpreters who were highly skilled at word-for-word translations. Another potential weakness of our study is that we relied on physicians' diagnoses of somatisation rather using more objective diagnostic criteria. However, our objective was not to verify patients' psychiatric diagnoses, but rather to identify patients whose physicians considered them to have medically unexplained symptoms, and to explore those patients' understanding of their symptoms.

We were also unable to explore how physicians' explanatory models influenced patients' narratives. As Groleau and Kirmayer point out [34], psychophysiological and sociophysiological models provide plausible medical explanations for most common somatic symptoms, and these models can provide a potential bridge to patients' culturally based explanations for their symptoms that link social context with bodily distress. When physicians validate the physical nature and social meaning of patients' suffering, patients are more likely to acknowledge the influence of psychosocial stress on their physical condition. On the other hand, when physicians narrowly psychologise their patients' distress, they tend to shift the responsibility for the unexplained to the patient [36]. Patients may respond by insisting on medical and social answers to their suffering that provide healing strategies outside themselves. Understanding how patients and physicians influence each others' explanatory models and illness narratives is important, but would require a more interactional analysis of patients' symptom attributions and clinical experiences, which is beyond the scope of the current study.

## Conclusion

The findings from this exploratory study suggest that these patients' illness perspectives are influenced by both pre- and post-migration life and embedded in a larger socio-political and legal context of asylum. Awareness of how physical, psychological, social and legal aspects intermingle may help physicians to be more open and receptive to the explanations given by patients as to the causes of their distress. In the context of asylum, physicians may need to address both patients' health and social issues in order to develop a trusting, therapeutic alliance [37].

## Competing interests

The author(s) declare that they have no competing interests.

## Authors' contributions

NJP and PH conceived and designed the study. NJP conducted the interviews. NJP and PH analyzed and interpreted the data and wrote the manuscript.

## Pre-publication history

The pre-publication history for this paper can be accessed here:


